# Travelling Editorials

**Published:** 1843-04

**Authors:** 


### THE WESTERN JOURNAL.

Vol. VII.—No. IV.

LOUISVILLE, APRIL 1, 1843.

### TRAVELLING EDITORIALS.

Already do we repent of our rash resolve, to take up, at stated periods, the pen editorial; and if said determination had not been made public, we should forthwith repeal it. Doubtless the *public*, that is our readers, would without much persuasion consent to such a repeal; but who is to move in the matter? One party is likely to be too proud—the other two courteous, and there the matter must rest.

### *Removal of Dr. Barton to Cuba.*

Two days after our arrival in New Orleans, Dr. E. H. Barton, formerly a Professor in the Medical College of Louisiana, and lately President of the Board of Health of the City, (both of which establishments he was mainly instrumental in effecting), departed for Havana. If not intending to expatriate himself, this gentleman, from circumstances in the health of his family, may sojourn there for some years, during which, we trust, he will make a thorough investigation of its fitness for our invalids in the winter. Dr. Barton's long practical acquaintance with the diseases of these climates, together with his science and urbanity, must render him a valuable physician and friend to such of our invalids as may visit the island of Cuba.

*Winter and Spring visits of Invalids to New Orleans.*

It is difficult to understand why physicians will advise their patients with pulmonary disease to come into Louisiana in the winter, and early spring. Nothing in general could be more exceptionable. It is nearly a month since we entered the limits of this State, during which there have, it is true, been a few fair days, but with one exception they were too cold for the valetudinary. The dampness of the atmosphere through the months of February and March, is so great as to render it injurious to all who labor under pulmonary disease; and the exposures on a winter voyage, are such as no invalid ought to suffer. They who seek a milder climate, should do so in November, and continue in it till after the vernal equinox.

*Winter Temperature of the Mississippi.*

The surface temperature of the Mississippi, from the mouth of the Ohio to the Balize, we have found to be, during February, from 34° to 44° of Fah. Difference of latitude about 8°—general course of the river South. Thus a degree of latitude raises the heat of the river a degree and a quarter. But the effect is not wholly ascribable to change of climate, but likewise to change of altitude; though the influence of the latter must be less than that of the former, as it does not, probably, exceed two hundred and fifty feet; or about thirty feet to the degree of latitude. The discharge, for three months, of so great a volume of cold water into the Gulf, must exert an influence on its temperature, which might be ascertained by a sufficient number of observations. We have as yet had opportunity to make a few only. In the "S.W. pass," beyond the bar, and within the geographical limits of the Gulf, we found the turbid and saltless river-water to be 44°, while at the depth of between fifty and sixty feet, where the water was nearly transparent, and decidedly salt, the heat was 51°. On one side of the fresh water, where the appearance was somewhat turbid and the taste brackish, the heat was 53°, and a little beyond, in the green and salt water, it was 56° and 57°.

*Medical College of Louisiana.*

We have made one visit to this Institution. Beginning its session later in autumn than the other schools of the Union, on account of the occasional prevalence of yellow-fever in November, the lectures continue through the month of March. We regret to say, however, that many of the pupils start home at the end of February. The number of the present session is, we understand, about thirty-five; which is an advance upon preceding years. They are principally from this State, Alabama, and Mississippi. The lectures are delivered in a rented house, but the Professors have begun the creation of a fund for the erection of an appropriate edifice. Neither the State nor the city has done any thing for the Institution. Its Professors are seven in number, who deliver from four to five lectures daily. The opportunities for practical anatomy are ample. The tickets of the Professors are twenty dollars each. It must be admitted that this school, in the number of its pupils, has fallen short of the expectations under which it was established; but we have not had an opportunity of investigating the causes which have retarded its growth; and which, it is to be hoped, may ere long be removed.

*Death of Tiger-Tail.*

In two visits to the New Orleans' barracks the head quarters of Brig. Gen. Arbuckle, we saw the Seminole Chief Tiger-Tail, on a sick bed. We were surprised to find him capable of conversing with us, but were told that before the war commenced, he had spent a year in the family of Gov. Duval, of Florida. At the times of our visits the captive warrior, labored under a fever, with cough, and a leg more or less swollen and inflamed from accidental injury. By auscultating his naked and weather-beaten chest we heard, what perhaps but few had ever heard, the palpitations of his savage but patriotic heart. Gen. Arbuckle, and the skilful surgeon of the post, Dr. Randall, were anxious that he should receive the treatment which his case required; but he preferred his own physician, under whose incantations he expired a few days since. Since that time, his surrendered countrymen have been sent on to Fort Gibson.

*New Orleans*, March 10, 1843. D.

